# Potential limitations of behavioral plasticity and the role of egg relocation in climate change mitigation for a thermally sensitive endangered species

**DOI:** 10.1002/ece3.4774

**Published:** 2019-01-28

**Authors:** Michael J. Liles, Tarla Rai Peterson, Jeffrey A. Seminoff, Alexander R. Gaos, Eduardo Altamirano, Ana V. Henríquez, Velkiss Gadea, Sofía Chavarría, José Urteaga, Bryan P. Wallace, Markus J. Peterson

**Affiliations:** ^1^ Asociación ProCosta San Salvador El Salvador; ^2^ Department of Communication, Environmental Science and Engineering Program University of Texas at El Paso El Paso Texas; ^3^ National Oceanic and Atmospheric Administration – National Marine Fisheries Service Southwest Fisheries Science Center La Jolla California; ^4^ Department of Biology San Diego State University San Diego California; ^5^ Fauna and Flora International Managua Nicaragua; ^6^ School of Earth, Energy & Environmental Sciences Stanford University Stanford California; ^7^ Conservation Science Partners, Inc. Fort Collins Colorado; ^8^ Nicholas School of the Environment Duke University Marine Lab Beaufort North Carolina; ^9^ Department of Biological Sciences University of Texas at El Paso El Paso Texas; ^10^ Eastern Pacific Hawksbill Initiative San Diego California

**Keywords:** egg relocation, environmental policy, mangrove estuaries, nest‐site selection, reproductive behavior, sand temperature, sea turtle, sea‐level rise, species redistribution, temperature‐dependent sex determination

## Abstract

Anthropogenic climate change is widely considered a major threat to global biodiversity, such that the ability of a species to adapt will determine its likelihood of survival. Egg‐burying reptiles that exhibit temperature‐dependent sex determination, such as critically endangered hawksbill turtles (*Eretmochelys imbricata*), are particularly vulnerable to changes in thermal regimes because nest temperatures affect offspring sex, fitness, and survival. It is unclear whether hawksbills possess sufficient behavioral plasticity of nesting traits (i.e., redistribution of nesting range, shift in nesting phenology, changes in nest‐site selection, and adjustment of nest depth) to persist within their climatic niche or whether accelerated changes in thermal conditions of nesting beaches will outpace phenotypic adaption and require human intervention. For these reasons, we estimated sex ratios and physical condition of hatchling hawksbills under natural and manipulated conditions and generated and analyzed thermal profiles of hawksbill nest environments within highly threatened mangrove ecosystems at Bahía de Jiquilisco, El Salvador, and Estero Padre Ramos, Nicaragua. Hawksbill clutches protected in situ at both sites incubated at higher temperatures, yielded lower hatching success, produced a higher percentage of female hatchlings, and produced less fit offspring than clutches relocated to hatcheries. We detected cooler sand temperatures in woody vegetation (i.e., coastal forest and small‐scale plantations of fruit trees) and hatcheries than in other monitored nest environments, with higher temperatures at the deeper depth. Our findings indicate that mangrove ecosystems present a number of biophysical (e.g., insular nesting beaches and shallow water table) and human‐induced (e.g., physical barriers and deforestation) constraints that, when coupled with the unique life history of hawksbills in this region, may limit behavioral compensatory responses by the species to projected temperature increases at nesting beaches. We contend that egg relocation can contribute significantly to recovery efforts in a changing climate under appropriate circumstances.

## 1 INTRODUCTION

Anthropogenic climate change is widely considered a major threat to global biodiversity (Foden et al., 2013; Parmesan & Yohe, 2003; Poloczanska et al., 2013), with 15%–37% of Earth's species potentially “committed to extinction” by 2050 (Thomas et al., 2004). The ability of a species to exhibit compensatory responses to climate‐driven environmental changes will determine its likelihood of survival; species more able to adjust to new environments or adapt to local climatic conditions will have a greater likelihood of persisting than those that cannot (Sinervo et al., 2010). Because the influence of climate change can vary among taxa and geographic regions (Parmesan, 2007), species may adapt in a variety of ways to mitigate unfavorable conditions (Bellard, Bertelsmeier, Leadley, Thuiller, & Courchamp, 2012), including evolutionary changes (Shefferson, Mizuta, & Hutchings, 2017) and spatiotemporal shifts in behavior (Chen, Hill, Ohlemüller, Roy, & Thomas, 2011; Yang & Rudolf, 2010).

However, life histories of some species may predispose them to higher levels of vulnerability than other species (Duputié, Rutschmann, Ronce, & Chuine, 2015). For example, ectotherms are particularly sensitive to changes in thermal regimes (Telemeco, Elphick, & Shine, 2009). In many reptiles, nest temperature regulates egg incubation duration, determines offspring sex, and affects progeny performance and survival (Bull, 1980; Van Damme, Bauwens, Braña, & Verheyen, 1992; Georges, 2013; Pike, 2014; Standora & Spotila, 1985). Adult female reptiles could respond to climate change by altering nesting range distribution, nesting phenology (i.e., timing of nesting), location of nest (e.g., amount of shade cover), and nest depth (Ewert, Lang, & Nelson, 2005; Pike, 2013b; Refsnider, Bodensteiner, Reneker, & Janzen, 2013; Schwanz & Janzen, 2008). For instance, maternal nest‐site choice can compensate for climatic variation among populations of the Australian water dragon (*Physignathus lesueurii*; Doody et al., 2006). Similarly, behavioral plasticity in painted turtles (*Chrysemys picta bellii*) can allow females to match shade cover over nests with prevailing environmental conditions to influence the sex ratio of offspring (Refsnider & Janzen, 2012).

Sea turtles are long‐lived, late‐maturing species that exhibit temperature‐dependent sex determination (TSD). Pivotal temperature (i.e., temperature that produces 50% of each sex; Yntema & Mrosovsky, 1980) is relatively conserved among sea turtle species and is centered within a transitional range of temperatures (TRT; ~1–3°C) that generally produce mixed sex ratios, where values above or below the narrow width of the TRT produce only one sex (Mrosovsky & Pieau, 1991; Wibbels, 2003). Successful egg development in sea turtles must occur between 25°C and 35°C (Ackerman, 1997), and temperature variations of ~1°C can markedly skew hatchling sex ratios (Mrosovsky, Kamel, Diez, & Dam, 2009). Most studies report female‐biased sex ratios (Hawkes, Broderick, Godfrey, & Godley, 2009; Wibbels, 2003), with some populations currently producing ≥90% female offspring (Broderick, Godley, Reece, & Downie, 2000; Godfrey, D'amato, Marcovaldi, & Mrosovsky, 1999; Marcovaldi, Godfrey, & Mrosovsky, 1997; Marcovaldi et al., 2014; Patino‐Martinez, Marco, Quinones, & Hawkes, 2012b). Climate models predict levels of warming between +1.6°C and +4.0°C for Central America by 2100 (Magrin, Marengo, & Boulanger, 2014), which would place additional thermal stress on embryonic development that may be nearing lethal thresholds with increasing frequency in many populations (Pike, 2014; Santidrián Tomillo et al., 2012; Valverde, Wingard, Gómez, Tordoir, & Orrego, 2010).

Given their complex life histories and reliance on marine and terrestrial habitats during their lifecycle, it is unclear how sea turtles will respond to climate‐driven change in these environments. Changes in nesting phenology of sea turtles have been observed in multiple locations worldwide (Dalleau et al., 2012; Neeman, Robinson, Paladino, Spotila, & O'connor, 2015; Weishampel, Bagley, Ehrhart, & Weishampel, 2010), and further shifts in global distributions of nesting are forecasted (Pike, 2013a, 2013b). Additionally, because TSD and thermal thresholds of embryonic development are highly conserved among sea turtle species (Davenport, 1997; Wibbels, 2003), female turtles could potentially alter nest depth or site on a beach to mitigate increased temperatures (Roosenburg, 1996). Regardless, whether behavioral plasticity in nesting will enable sea turtles to meet the challenges posed by climate change remains uncertain (Hamann et al., 2010; Hawkes, Broderick, Godfrey, & Godley, 2007).

Given potential limitations of plastic compensatory responses of sea turtles to accelerated changes in thermal conditions of nesting beaches, it is possible that sea turtles will be unable to adapt quickly enough to offset negative consequences to population demographics. In such cases, human intervention may be required to ensure population persistence. Relocation of sea turtle eggs as a management strategy used to increase hatchling production and enhance population recovery is ubiquitous worldwide (Chacón‐Chaverri & Eckert, 2007; Formia, Tiwari, Fretey, & Billes, 2003; García, Ceballos, & Adaya, 2003; Naro‐Maciel, Mrosovsky, & Marcovaldi, 1999; Patino‐Martinez, Marco, Quinones, & Hawkes, 2012b). By utilizing internationally recognized best practices throughout the egg relocation process (Eckert et al., 1999), many of the concerns about possible undesired biological outcomes (Mrosovsky, 2006; Pilcher & Enderby, 2001; Prichard, 1980) can be avoided or mitigated (Kornaraki, Matossian, Mazaris, Matsinos, & Margaritoulis, 2006; Marcovaldi & Marcovaldi, 1999; Patino‐Martinez, Marco, Quinones, Abella, et al., 2012a). Because temperatures are predicted to increase substantively in Central America over a relatively short period, the influence of sea turtle egg relocation on the thermal regimes of nest environments, primary sex ratios, and hatchling fitness compared with in situ clutches is a top research priority, particularly for severely depleted populations of highly endangered species.

Critically endangered hawksbill turtles (*Eretmochelys imbricata*) in the eastern Pacific Ocean belong to one of the least resilient (Fuentes, Pike, Dimatteo, & Wallace, 2013) and most threatened marine turtle regional management units (RMU) in the world (Wallace et al., 2011), with fewer than 700 adult females nesting along 15,000 km of Latin American coastline (Gaos et al., 2017). Further, >70% of this nesting activity is concentrated on low‐relief beaches in mangrove estuaries at Bahía de Jiquilisco in El Salvador and Estero Padre Ramos in Nicaragua (Gaos et al., 2017; Liles, Peterson, Seminoff, et al., 2015b)—ecosystems that are particularly vulnerable to increasing global temperatures and sea‐level rise (Gilman, Ellison, Duke, & Field, 2008).

**Figure 1:**
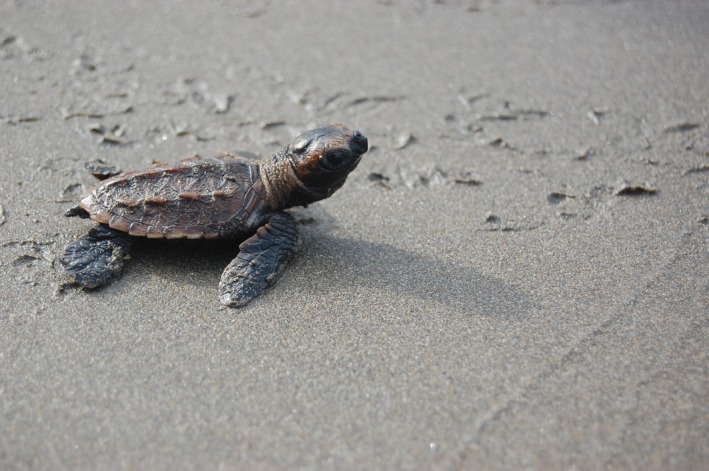
Recently emerged hatchling hawksbill turtle released from a hatchery at Bahía de Jiquilisco, El Salvador

In this study, we investigated whether behavioral plasticity in this species is likely to be able to compensate for projected climate change and what the role of egg relocation may be as a mitigation strategy. The objectives of our study were to (a) estimate sex ratios and physical condition of hatchling hawksbills under natural and manipulated conditions (Figure 1) and (b) generate and analyze thermal profiles of nest environments. Our results provide the first empirical assessment of the efficacy of nest protection strategies for this severely depleted RMU. Based on our findings, we offer recommendations for mitigation strategies that complement potential plastic adaptive responses to climate change demonstrated by nesting hawksbills in mangrove ecosystems.

## 2 MATERIALS AND METHODS

**Figure 2:**
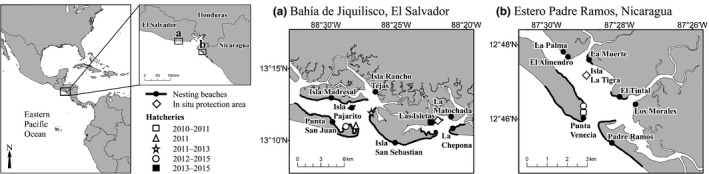
Locations of hawksbill nesting beaches, hatcheries, and in situ nest protection areas at (a) Bahía de Jiquilisco, El Salvador (2011–2015) and (b) Estero Padre Ramos, Nicaragua (2010–2015)

Our study was conducted at Bahía de Jiquilisco (13°13′N, 88°32′W) in El Salvador and Estero Padre Ramos (12°48′N, 87°28′W) in Nicaragua, which are located on the western and eastern borders of Gulf of Fonseca on the Pacific coast of Central America, respectively (Figure 2). Hawksbill nesting occurs primarily during the rainy season between May and September, with a peak in June and July. Contrary to typical contiguous open‐coast beaches used by nesting hawksbills in other oceanic regions (Loop, Miller, & Limpus, 1995; Mrosovsky, 2006), hawksbills at these two sites nest on low‐relief beaches scattered within mangrove estuaries (Gaos et al., 2017; Liles, Peterson, Seminoff, et al., 2015b).

Bahía de Jiquilisco is located on the south‐central coast of El Salvador and has hawksbill nesting habitat (42.1 km) comprised of eight distinct fine‐grained sand beaches with three hatcheries and one in situ nest protection area (Figure 2). A fragmented mosaic of second‐growth coastal forest and small‐scale fruit tree plantations 10–15 m wide from the high water line is present at most nesting beaches (Liles, Peterson, Seminoff, et al., 2015b). Moderate development exists in some nesting areas, particularly along eastern and western Punta San Juan, eastern and western Isla Madresal, and northern Isla San Sebastian.

Estero Padre Ramos is situated on the northwestern Pacific coast of Nicaragua and consists of eight distinct fine‐grained sand beaches (12.8 km), with one hatchery and one in situ nest protection area (Figure 2). Intact secondary coastal forest extends >100 m landward from the high water line at most beaches (Liles, Peterson, Seminoff, et al., 2015b). Nesting areas have experienced relatively minimal development, with Padre Ramos most impacted.

### 2.1 Nest distribution and protection strategies

Beach patrols were conducted from 1 April to 15 October 2011–2015 at Bahía de Jiquilisco and 1 May to 15 October 2010–2015 at Estero Padre Ramos. Project personnel and a network of >200 trained local egg collectors monitored nesting habitat continually from 18:00 to 06:00 daily by foot and boat in search of female hawksbills (~50% detection) and nests at both sites. Because of depressed socioeconomic conditions of coastal communities in both countries, local residents consider hawksbill eggs an economic resource, resulting in collection of nearly 100% of eggs (Liles et al., 2016; Liles, Peterson, Lincoln, et al., 2015a). Consequently, conservation organizations purchase eggs encountered and/or collected by local residents for protection to prevent their sale for human consumption. Each nesting season ~10% of hawksbill clutches typically are protected in situ via agreements with local residents to leave eggs in place, and ~90% of clutches are relocated to nearby areas of the beach or to hatcheries to avoid human depredation (Liles et al., 2016; Liles, Peterson, Lincoln, et al., 2015a).

The protection strategy employed for encountered nests depended on the likelihood of predation at the original site of egg deposition, the location of the nest, and the year at each site. At Bahía de Jiquilisco during 2011–2015, clutches deposited at the in situ protection area were not manipulated; however, we buried a surface‐enclosed wire mesh cylinder (diameter, 50 cm; height, 60 cm) around each clutch at a depth of ~55 cm after oviposition had completed to reduce the probability of egg predation; we removed this barrier three days prior to the estimated date of hatching or when a depression in the sand was observed. Clutches deposited at beaches ≤3 km from the in situ protection area were relocated to the in situ protection area for protection, except during 2013–2015, when clutches were relocated to a hatchery. We relocated remaining clutches to the nearest hatchery, depending on the location of deposition (Table 1). At Estero Padre Ramos during 2010–2015, we did not manipulate clutches deposited at the in situ protection area. We relocated all clutches deposited at other beaches to a hatchery (Table 1), except during 2010 and 2011, when some clutches were relocated to an area of beach near the hatchery because the hatchery had reached capacity or was not yet operational.

**Table 1:** Hatchery and shading characteristics at Bahía de Jiquilisco, El Salvador, 2011–2015, and Estero Padre Ramos, Nicaragua, 2010–2015

Site Hatchery	Year	Size (m^2^)	Shade (%)	Shading materials
Bahía de Jiquilisco				
Punta San Juan	2011	100	50	PL
	2012–2014	100	96.3	SC; FC
	2015	100	100	SC; FC
La Pirraya	2011	50	70	SC
	2012–2013	50	84.5	SC; FC
Las Isletas	2013–2014	50	90.1	SC; FC
	2015	50	100	SC; FC
Estero Padre Ramos				
Punta Venecia	2010–2011	125	100	SC; FC
	2012–2015	125	77.7	SC; FC

Note

FC: forest canopy; PL: palm leaves; SC: shade cloth.

For clutches relocated on the beach or to a hatchery, we measured the dimensions of original nest cavities and attempted to emulate these dimensions in artificial nests. We relocated most clutches <12 hr after deposition to minimize movement‐induced mortality during transfer and reburial (Limpus, Baker, & Miller, 1979).

### 2.2 Hatchling sex ratios and physical condition

Although direct methods for estimating hatchling sex ratios, such as histological evaluation of gonads, are highly accurate for sexing individual hatchlings, they are logistically infeasible to perform on endangered species. Indirect methods—including nest temperature and incubation duration—are reliable proxies when direct methods are infeasible (Wibbels, 2003). Because financial and logistical constraints prohibited us from recording nest temperatures at Estero Padre Ramos in 2010–2011, we used incubation duration values obtained for offspring‐producing nests to estimate primary sex ratios at both sites to provide results that are commensurable across sites and among years.

We used published data for hawksbills that related incubation duration to sex ratio based on constant temperature incubator experiments to convert the incubation duration of each clutch into hatchling sex ratio (Godfrey et al., 1999). For incubation duration calculations, the incubation period was calculated as the number of days between the date and hour of clutch deposition and the date and hour of first hatchling emergence. For nests where the date of emergence was unavailable (*n* = 50 nests, 2.5% of total) or where no hatchlings emerged but were found alive during exhumation (*n* = 30 nests, 1.5% of total), we used the average incubation duration of the nest protected using the same strategy immediately before and after the nest without date of emergence or with live hatchlings that did not emerge. We used a one‐ to four‐day correction factor for the hatching‐to‐emergence interval in overall hatchling sex ratio calculations to establish a range of mean values that accounts for potential differences in the amount of time it takes a hatchling to emerge from the nest after hatching, which would affect incubation duration estimates (Godfrey et al., 1999; Godfrey & Mrosovsky, 1997). We calculated the overall sex ratio for each protection strategy within and across sites, and among years, and for specific comparisons among nest protection strategies and between sites, we used a three‐day correction factor based on nests that showed a marked temperature signal at hatching (mean = 2.9 ± 0.2 days, *n* = 3; King, Cheng, Tseng, Chen, & Cheng, 2013).

Hawksbill nests at Bahía de Jiquilisco and Estero Padre Ramos hatch about 55–70 days after egg deposition. For clutches protected during the nesting season, successful nests were excavated within 48 hr of first hatchling emergence to evaluate hatching success and failed nests were excavated on day 70 of incubation to identify potential causes of nest failure. We recorded the following metrics for reproductive output and hatchling physical condition for each clutch: size (i.e., total number of eggs), hatching success (i.e., proportion of eggs that produced live hatchlings that emerged or were found in the nest during exhumation), and straight carapace length of hatchlings measured with calipers (Bahía de Jiquilisco, Neiko Tools, Taiwan; Estero Padre Ramos, Wilmar Corp, Tukwila, WA, USA) and hatchling mass using a digital scale (Bahía de Jiquilisco, American Weigh Scales, Norcross, GA, USA) and a spring scale (Estero Padre Ramos, Wilmar Corp, Tukwila, WA, USA).

### 2.3 Thermal profiles of sand and nests

To measure intrabeach variation in temperature during the hawksbill nesting season, we divided the beach into four zones from ocean to forest, based on vegetative cover: (a) open sand (no vegetation), (b) nonwoody vegetation (herbaceous vegetation), (c) woody vegetation border (near the forest or plantations, but not completely surrounded by trees), and (d) woody vegetation (surrounded by trees; Liles, Peterson, Seminoff, et al., 2015b). We buried HOBO U22 data loggers (Water Temp Pro v2, Onset Computer Corporation, Bourne, MA, USA) in each of the four beach zones at two sand depths (30 and 60 cm), which are near the upper and lower range of hawksbill nest depths, respectively (Kamel & Mrosovsky, 2006a). At Bahía de Jiquilisco during 2012–2015, data loggers (hereafter referred to as “loggers”) were buried in beach zones along three transects, each separated by 500 m. Not all beach zones were present along each transect, which resulted in one or two paired‐logger sites per zone per year. At Estero Padre Ramos during 2015, loggers were buried in four beach zones (*n* = 1 paired‐logger site per zone). To assess the effects of deforestation on thermal conditions of nesting beaches (Kamel & Mrosovsky, 2006a), we placed loggers in areas cleared of vegetation at Bahía de Jiquilisco in 2012–2015 (*n* = 3 paired‐logger sites per year) and at Estero Padre Ramos in 2015 (*n* = 1 paired‐logger site). Loggers had an accuracy of ±0.2°C (per manufacturer specifications) and recorded the temperature every 30 min. We averaged recorded values to give a mean daily temperature for each logger, which facilitated comparisons with previous studies (e.g., Glen & Mrosovsky, 2004; Kamel & Mrosovsky, 2006b; Hawkes et al., 2007). Loggers that were stolen (*n* = 4 at Estero Padre Ramos), lost due to beach erosion (*n* = 4 at Bahía de Jiquilisco), or did not function properly during data collection (*n* = 4 at Bahía de Jiquilisco) were excluded from analyses. The stolen loggers at Estero Padre Ramos resulted in loss of temperature data for the open sand zone and deforested area.

To protect hawksbill clutches deposited on beaches where in situ protection and relocation on the beach were infeasible, shaded hatcheries were constructed at nesting beaches at both sites that typically operated from 1 May to 31 October annually and whose dimensions varied according to the capacity required for relocated clutches (Table 1). We buried loggers in the center of each hatchery at the two depths at Bahía de Jiquilisco in 2012–2015 (*n* = 2 or 3 hatcheries) and at Estero Padre Ramos in 2015 (*n* = 1 hatchery; Table 1). Temperature was recorded every 30 min and then averaged to obtain a mean daily temperature for each logger. Loggers that malfunctioned during data collection (*n* = 2 at Bahía de Jiquilisco) were not included in analyses.

To measure temperature in hawksbill nests during the incubation period, we placed HOBO U22 or HOBO U23 (Pro v2 Temperature/Relative Humidity, Onset Computer Corporation, Bourne, MA, USA) loggers in the center of the egg mass of clutches incubated in situ, relocated on the beach, and in hatcheries at Bahía de Jiquilisco during 2011–2015 and Estero Padre Ramos during 2012–2015. Deployment of loggers was spread across the nesting season to represent the temporal distribution of nests (*n* = 2 to 14 nests per month per site). Loggers recorded the temperature at 2.5‐min or at 5‐min intervals, depending on the logger model, and remained in the nest during the entire incubation period until they were removed at post‐hatching nest excavation. We calculated daily mean temperature for each logger, which was then used to calculate the mean nest temperature during the entire incubation period and the mean nest temperature for the middle third of incubation when offspring sex is determined (i.e., thermosensitive period; Rimblot, Fretey, Mrosovsky, Lescure, & Pieau, 1985).

### 2.4 Shade cover in hatcheries

At Bahía de Jiquilisco (2011–2015) and Estero Padre Ramos (2010–2015), we shaded nests in hatcheries using a variety of methods that included palm leaves, shade cloth (Bahía de Jiquilisco: Saran Verde, Freund, San Salvador, El Salvador, 75% radiation block; Estero Padre Ramos: undetermined model, 75% radiation block), and natural forest canopy (Table 1). Shade cover from palm leaves and forest canopy over hatcheries was measured using a convex spherical densitometer (Ben Meadows, Janesville, WI, USA), except at Punta San Juan hatchery at Bahía de Jiquilisco in 2011 and Estero Padre Ramos in 2010–2011 (Figure 2), where palm leaf cover above nests was estimated and complete forest cover over the hatchery effectively represented 100% shading, respectively. The same shade cover value was used for a hatchery across years when it remained in same location as the previous year and no changes were made to the forest canopy nor the shade cloth.

### 2.5 Statistical analyses

We used version 4.0.3 of Girondot's (1999) method to convert incubation duration of hawksbill clutches protected at our sites into hatchling sex ratios. Two‐way analysis of variance (ANOVA) was used to test for differences among the three nest protection strategies in each of 10 parameters of incubation regime (i.e., nest temperature—minimum, maximum, mean of entire period, mean of thermosensitive period—during incubation, incubation duration, and nest depth) and hatchling condition (i.e., hatching success, offspring sex ratios, hatchling mass, and hatchling length) at Bahía de Jiquilisco and Estero Padre Ramos, and among years. We also used a two‐way ANOVA to test for differences in sand temperature within and among the six nest environments between logger depths and years at Bahía de Jiquilisco and within and among the four nest environments between logger depths at Estero Padre Ramos. For summary statistics, values are expressed as mean ± *SD*. We computed all analyses using JMP Pro 12.0.0 (SAS Institute, Cary, NC, USA), with an alpha level of 0.05 where relevant.

## 3 RESULTS

### 3.1 Nest distribution and protection strategies

**Figure 3:**
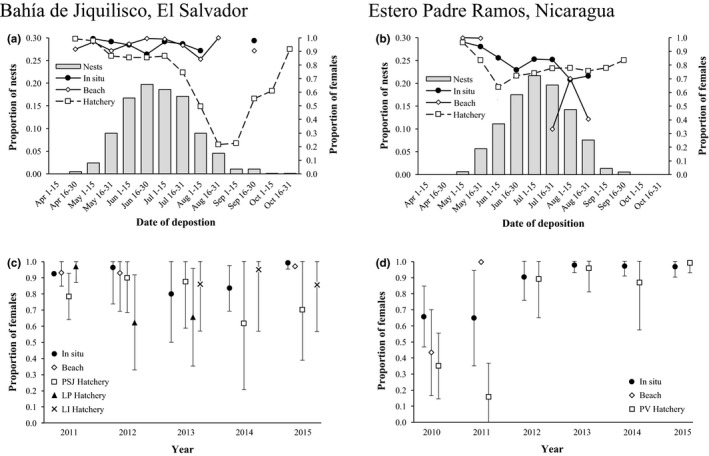
Estimated hawksbill hatchling sex ratios at Bahía de Jiquilisco, El Salvador (2011–2015) and Estero Padre Ramos, Nicaragua (2010–2015). (a, b) Bimonthly frequency distribution of hawksbill nesting (gray bars) and estimated offspring sex ratios from three nest protection strategies (lines) at Bahía de Jiquilisco, (*n* = 835 clutches) and Estero Padre Ramos, (*n* = 1,196 clutches), respectively. (c, d) Annual mean (±*SD*) estimated offspring sex ratios from each nest protection strategy at Bahía de Jiquilisco and Estero Padre Ramos, respectively

We recorded 2,154 nesting events from a minimum of 366 individual hawksbills, representing 72.8% of total nests recorded in the eastern Pacific during 2010–2015 and 69.3% of total mature females identified in the entire eastern Pacific region (Gaos et al., 2017). Of these nests, 877 (40.7%) were located at Bahía de Jiquilisco (2011–2015) and 1,277 (59.3%) at Estero Padre Ramos (2010–2015). Most hawksbills nested between May and August at Bahía de Jiquilisco (96.4%, *n* = 845 clutches) and Estero Padre Ramos (96.3%, *n* = 1,230 clutches), with a peak in nesting occurring in June and July (Bahía de Jiquilisco, 69.9%, *n* = 613 clutches; Estero Padre Ramos, 68.1%, *n* = 869 clutches; Figure 3a,b).

**Figure 4:**
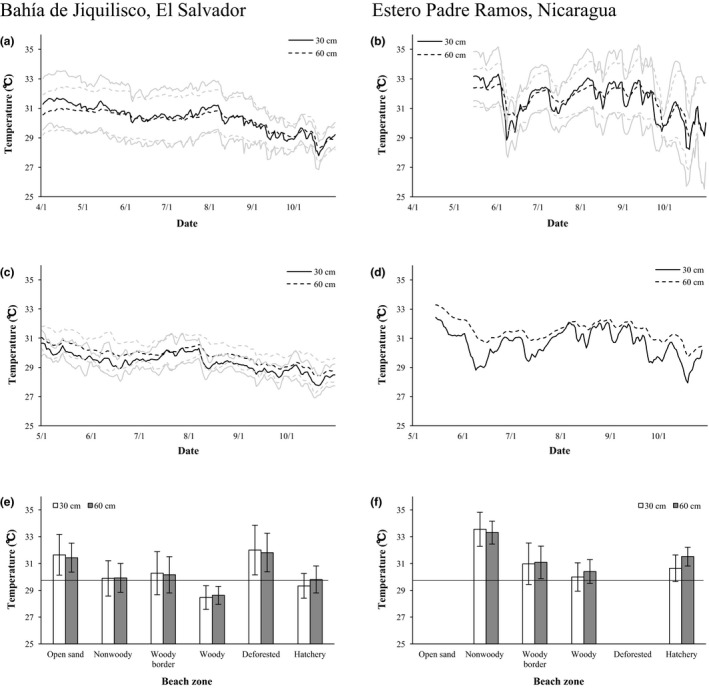
Sand temperature at two sand depths (30 and 60 cm) in hawksbill nest environments at Bahía de Jiquilisco, El Salvador (2012–2015) and Estero Padre Ramos, Nicaragua (2015). (a, b) Daily sand temperature (black lines; ±*SD*, gray lines) pooled across four and three beach zones over the hawksbill nesting season at Bahía de Jiquilisco, 1 April–31 October (*n* = 4,482 days) and Estero Padre Ramos, 15 May–31 October (*n* = 510), respectively. (c, d) Daily sand temperature (black lines; ±*SD*, gray lines) in hatcheries over the hawksbill nesting season at Bahía de Jiquilisco, 1 May–31 October (*n* = 1,514 days) and Estero Padre Ramos, 15 May–31 October (*n* = 170 days), respectively. (e and f) Sand temperature (mean ± *SD*) in six nest environments at Bahía de Jiquilisco (open sand, *n* = 1,481 days; nonwoody, *n* = 853; woody border, *n* = 1,706; woody, *n* = 851; deforested, *n* = 2,558; hatchery, *n* = 1,514) and in four nest environments at Estero Padre Ramos, (nonwoody, *n* = 170 days; woody border, *n* = 170; woody, *n* = 170; hatchery, *n* = 170), respectively. The horizontal black lines indicate the most conservative male‐producing pivotal temperature estimate for hawksbills among studied hawksbill populations (29.7°C; Godfrey et al., 1999).

Of 2,154 hawksbill clutches deposited at Bahía de Jiquilisco and Estero Padre Ramos, we protected 94.6% (*n* = 2,038) at both sites—153 (7.5%) were protected in situ, 123 (6.0%) relocated on the beach, and 1,758 (86.3%) relocated to hatcheries, with the remaining four clutches (0.2%) protected using other methods and not included in this study. The remaining 5.4% of clutches were depredated by humans or domestic animals. We recorded the temperature of 23 (39.7%) and 21 (22.6%) clutches protected in situ, 12 (21.1%) and 0 (0.0%) clutches relocated on the beach, and 144 (17.2%) and 74 (7.3%) clutches relocated to hatcheries at Bahía de Jiquilisco and Estero Padre Ramos, respectively.

### 3.2 Incubation regime

Across sites, mean nest depth was 40.3 ± 4.7 cm (range = 27–61, *n* = 1,836 nests), with shallower depth in clutches protected in situ (39.3 ± 4.0 cm, *n* = 108) and relocated on the beach (39.2 ± 5.1, *n* = 71) than in hatcheries (40.4 ± 4.7 cm, *n* = 1,657). Nest depth was shallower at Bahía de Jiquilisco than Estero Padre Ramos (Table 2), with significant differences among protection strategies at both sites (Table 3).

**Table 2:** Values (±*SD*) for 10 parameters of incubation regime and hatchling condition for each of three hawksbill nest protection strategies at Bahía de Jiquilisco, El Salvador (2011–2015), and Estero Padre Ramos, Nicaragua (2010–2015)

Site strategy	Incubation regime^a^						Hatchlings			
	Min temp (°C)	Max temp (°C)	Mean temp (°C)	Mean temp, TSP^b^ (°C)	Duration (days)	Nest depth^a^ (cm)	Hatching^c^ (%)	Female^a^, ^d^ (%)	Mass^c^ (g)	Length^c^ (cm)
Bahía de Jiquilisco										
In situ	26.9 ± 1.7 (23)	34.6 ± 1.3 (23)	30.9 ± 1.1 (23)	30.6 ± 1.4 (23)	54.3 ± 3.9 (44)	38.2 ± 3.2 (58)	37.7 ± 31.1 (58)	88.9–96.2 (44)	10.78 ± 1.23 (625)	3.75 ± 0.15 (601)
Relocated on beach^e^	26.6 ± 0.7 (12)	35.5 ± 1.9 (12)	31.0 ± 0.1 (12)	30.6 ± 1.0 (12)	55.5 ± 3.7 (34)	36.7 ± 3.3 (46)	39.5 ± 32.2 (46)	84.5–95.5 (34)	11.44 ± 1.22 (420)	3.75 ± 0.17 (491)
Hatchery	26.9 ± 1.3 (144)	33.7 ± 1.7 (144)	30.2 ± 1.1 (144)	29.8 ± 1.1 (144)	58.4 ± 4.6 (627)	38.7 ± 3.2 (731)	55.8 ± 33.1 (731)	66.9–83.9 (627)	11.19 ± 1.22 (10,930)	3.76 ± 0.19 (9,355)
Total	26.9 ± 1.3 (179)	34.0 ± 1.7 (179)	30.3 ± 1.1 (179)	29.9 ± 1.2 (179)	58.0 ± 4.7 (705)	38.5 ± 3.2 (835)	53.8 ± 33.4 (835)	68.6–84.9 (705)	11.18 ± 1.23 (11,975)	3.76 ± 0.19 (10,447)
Estero Padre Ramos										
In situ	27.6 ± 1.5 (21)	34.1 ± 1.2 (21)	30.8 ± 0.9 (21)	30.7 ± 1.2 (21)	57.2 ± 3.5 (91)	40.5 ± 4.5 (50)	50.2 ± 28.1 (93)	68.1–88.3 (91)	11.21 ± 1.29 (448)	3.66 ± 0.17 (386)
Relocated on beach^f^	–	–	–	–	61.3 ± 4.3 (68)	43.8 ± 4.9 (25)	50.3 ± 31.2 (77)	32.3–58.7 (68)	12.31 ± 1.53 (807)	3.68 ± 0.16 (837)
Hatchery	27.8 ± 1.4 (74)	33.5 ± 1.4 (74)	30.6 ± 1.0 (74)	30.3 ± 1.0 (76)	57.7 ± 5.7 (981)	41.7 ± 5.2 (926)	61.1 ± 26.8 (1,026)	66.0–78.8 (981)	11.88 ± 1.52 (15,741)	3.70 ± 0.18 (15,791)
Total	27.8 ± 1.4 (95)	33.7 ± 1.4 (95)	30.7 ± 0.1 (95)	30.3 ± 1.1 (97)	57.9 ± 5.6 (1,140)	41.7 ± 5.2 (1,001)	59.6 ± 27.5 (1,196)	64.2–78.3 (1,140)	11.88 ± 1.52 (16,996)	3.70 ± 0.18 (17,014)

a Values in parentheses represent the number of clutches.

b TSP, thermosensitive period.

c Values in parentheses represent the number of hatchlings.

d Range of mean values including 1‐ to 4‐day correction factor for hatchling emergence.

e Excludes year 2013–2014.

f Excludes years 2012–2015.

**Table 3:** Two‐way ANOVA results for differences in each of six incubation regime variables among three nest protection strategies (in situ, relocated on beach, and hatchery) among years at Bahía de Jiquilisco, El Salvador (2011–2015), and Estero Padre Ramos, Nicaragua (2010–2015)

Incubation regime source	Bahía de Jiquilisco					Estero Padre Ramos				
	*df*	SS	MS	*F*	*p*	*df*	SS	MS	*F*	*p*
Minimum temperature										
Strategy	2	0.0134	0.0067	0.0038	0.9620	1	0.0068	0.0068	0.0039	0.9501
Year	4	13.2291	3.3073	1.8711	0.1177	3	36.6277	12.2092	7.1135	0.0002
Error	171	302.2566	1.7676			90	154.4700	1.7163		
Total	177	316.3711	1.7874			94	192.0848	2.0435		
Maximum temperature										
Strategy	2	38.4874	19.2437	7.4344	0.0008	1	6.0188	6.0188	3.3209	0.0717
Year	4	30.4110	7.6028	2.9372	0.0221	3	10.0939	3.3647	1.8564	0.1427
Error	171	442.6272	2.5885			90	163.1187	1.8124		
Total	177	520.3395	2.9398			94	177.7459	1.2526		
Mean temperature										
Strategy	2	17.6325	8.8162	8.1319	0.0004	1	1.9680	1.9679	2.7767	0.0991
Year	4	7.7559	1.9390	1.7885	0.1333	3	27.1719	9.0573	12.7796	<0.0001
Error	171	185.3908	1.0842			90	63.7860	0.7087		
Total	177	210.7523	1.1907			94	91.6039	0.9745		
Mean temperature, TP										
Strategy	2	19.4525	9.7262	7.1573	0.0010	1	5.8476	5.8476	6.5880	0.0119
Year	4	6.3286	1.5822	1.1643	0.3284	3	28.2800	9.4267	10.6203	<0.0001
Error	171	232.3751	1.3590			90	79.8848	0.8876		
Total	177	259.4069	1.4656			94	111.3887	1.18499		
Incubation duration										
Strategy	2	726.7760	363.3880	18.3004	<0.0001	2	513.6550	256.8275	20.6619	<0.0001
Year	4	532.8525	133.2131	6.7087	<0.0001	5	20,192.0880	4,038.4176	324.8930	<0.0001
Error	694	13,800.4950	19.8568			1,132	14,070.7530	12.4300		
Total	701	15,228.9990	21.7247			1,139	35,122.7930	30.8365		
Nest depth										
Strategy	2	209.5189	104.7594	10.1693	<0.0001	2	251.7260	125.8630	8.0948	0.0003
Year	4	72.9575	18.2394	1.7705	0.1327	5	11,188.3430	2,237.6686	143.9148	<0.0001
Error	828	8,529.6924	10.3016			993	15,439.7250	15.5486		
Total	834	8,774.2069	10.5206			1,000	26,804.0230	26.8040		

Overall, mean nest temperature during the entire incubation period was 30.4 ± 1.1°C (*n* = 274 clutches), with slightly higher temperatures in clutches protected in situ (30.7 ± 1.0°C, *n* = 44) than clutches relocated to hatcheries (30.3 ± 1.1°C, *n* = 218). Mean nest temperature during the middle third of the incubation period was likewise higher in clutches protected in situ (30.6 ± 1.3°C, *n* = 44) than clutches relocated to hatcheries (29.9 ± 1.1°C, *n* = 218; overall, 30.1 ± 1.2°C, *n* = 276). There was little difference in nest temperature between sites (Table 2), but significant differences among nest protection strategies at both sites (Table 3).

Mean incubation duration was 57.9 ± 5.2 days (*n* = 1,845 clutches) across sites, with shorter duration of clutches protected in situ (56.3 ± 3.9 days, *n* = 135) than those relocated on the beach (59.4 ± 4.9 days, *n* = 102) and in hatcheries (58.0 ± 5.3 days, *n* = 1,608). Incubation duration was similar at Bahía de Jiquilisco and Estero Padre Ramos (Table 2), but significant differences existed among protection strategies and years at both sites (Table 3). At Bahía de Jiquilisco, incubation duration was significantly shorter (*t* = 9.8898, *df* =703, *p* < 0.0001) during the first half (1 April–15 July; 56.9 ± 3.6 days, *n* = 495 clutches) than the second half (16 July–31 October; 60.5 ± 5.7 days, *n* = 210) of the nesting season, whereas at Estero Padre Ramos, only marginal differences were detected (*t* = 0.9564, *df* = 1,138, *p* = 0.3391; first half, 57.8 ± 5.7 days, *n* = 656; second half, 58.1 ± 5.4 days, *n* = 484).

### 3.3 Hatchling production, sex ratios, and physical condition

Across sites, protected clutches had a mean size of 160.2 ± 37.8 eggs (range = 3–274, *n* = 2,031), a mean hatching success of 56.8 ± 30.2% (*n* = 2031), and produced 185,922 hatchlings. Hatching success was lower in clutches protected in situ (43.6 ± 30.0%, *n* = 153) than those relocated on the beach (46.2 ± 32.2%, *n* = 124) and in hatcheries (58.6 ± 29.7%, *n* = 1757). There were larger clutches and lower hatching success at Bahía de Jiquilisco than Estero Padre Ramos (Table 2), with significant differences in hatching success among nest protection strategies and years at both sites (Table 4). At Bahía de Jiquilisco, hatching success was significantly higher (*t* = 2.4390, *df* =833, *p* = 0.0149) during the first half (55.7 ± 31.8%, *n* = 559) than the second half (49.7 ± 35.8%, *n* = 276) of the nesting season, whereas at Estero Padre Ramos, it was only slightly higher (*t* = 1.3734, *df* = 1,197, *p* = 0.1699; first half, 60.6 ± 26.8%, *n* = 679; second half, 58.4 ± 28.5%, *n* = 520).

**Table 4:** Two‐way ANOVA results for differences in each of four hatchling condition variables among three nest protection strategies (in situ, relocated on beach, and hatchery) among years at Bahía de Jiquilisco, El Salvador (2011–2015), and Estero Padre Ramos, Nicaragua (2010–2015)

Hatchling condition source	Bahía de Jiquilisco					Estero Padre Ramos				
	*df*	SS	MS	*F*	*p*	*df*	SS	MS	*F*	*p*
Hatching success										
Strategy	2	2.1532	1.0766	11.2887	<0.0001	2	1.5695	0.7848	10.7819	<0.0001
Year	4	11.3926	2.8481	29.8639	<0.0001	5	1.7672	0.3534	4.8559	0.0002
Error	827	78.9171	0.0954			1,188	86.4682	0.0728		
Total	834	93.1719	0.1117			1,195	90.0317	0.0753		
Female hatchlings										
Strategy	2	1.2751	0.6375	7.3120	0.0007	2	4.1300	2.0650	42.2478	<0.0001
Year	4	2.9112	0.7278	8.3473	<0.0001	5	84.6158	16.9232	346.2371	<0.0001
Error	698	60.8585	0.0872			1,132	55.3292	55.3292		
Total	704	65.5458	0.0931			1,139	144.9736	0.1273		
Hatchling length										
Strategy	2	0.4086	0.2043	6.0778	0.0023	2	0.4543	0.2271	7.9771	0.0003
Year	4	7.2448	1.8112	53.8863	<0.0001	5	51.5192	10.3038	361.8605	<0.0001
Error	10,440	350.9056	0.0336			17,006	484.2393	0.0285		
Total	10,446	358.3552	0.0343			17,013	536.5475	0.0315		
Hatchling mass										
Strategy	2	127.6789	63.8395	46.1007	<0.0001	2	129.2909	64.6455	32.2017	<0.0001
Year	4	1,339.9367	334.9842	241.9039	<0.0001	5	5,035.8213	1,007.1643	501.6963	<0.0001
Error	11,968	16,573.0700	1.3848			16,988	34,103.7110	2.0075		
Total	11,974	18,044.3540	1.5070			16,995	39,496.0340	2.3240		

The overall range of means for the percentage of female hatchlings produced from protected clutches was 66.0 ± 37.6 to 81.0 ± 37.6% (*n* = 1,845), with a greater percentage of female hatchling production from clutches protected in situ (86.9 ± 20.1%, *n* = 135) than those relocated on the beach (63.2 ± 35.2%, *n* = 102) and in hatcheries (76.4 ± 34.4%, *n* = 1608). Of the hatchlings produced at both sites, there was a higher percentage of females at Bahía de Jiquilisco than Estero Padre Ramos (Table 2), with significant differences among protection strategies and years at both sites (Table 4). No correlation existed between male hatchling production and nest depth for clutches protected in situ (*r* ^2^ = 0.01, *F*_1,92_ = 1.16, *p* = 0.2840). At Bahía de Jiquilisco, the percentage of female hatchlings produced was significantly higher (*t* = 10.3636, *df* = 705, *p* < 0.0001) during the first half (87.3 ± 23.8%, *n* = 495) than the second half (63.1 ± 36.7%, *n* = 210) of the nesting season, whereas at Estero Padre Ramos, there were negligible differences (*t* = 0.0903, *df *= 1,138, *p* = 0.9281; first half, 73.7 ± 36.7%, *n* = 656; second half, 73.5 ± 34.9%, *n* = 484).

Hatchlings had a mean carapace length of 3.72 ± 0.18 cm (*n* = 27,461) and mean body mass of 11.59 ± 1.45 g (*n* = 28,971) across sites. Hatchling length was nearly identical among protection strategies (in situ, 3.71 ± 0.16 cm, *n* = 987; relocated on beach, 3.71 ± 0.17 cm, *n* = 1,277; hatchery, 3.72 ± 0.18 cm, *n* = 25,146), but hatchling mass was less in clutches protected in situ (10.96 ± 1.27 g, *n* = 1,073) than those relocated on the beach (12.01 ± 1.49 g, *n* = 1,227) and in hatcheries (11.60 ± 1.45 g, *n* = 26,670). Hatchlings produced at Bahía de Jiquilisco were slightly larger but weighed less than at Estero Padre Ramos (Table 2), and there were significant differences in hatchling length and mass among strategies and years at both sites (Table 4).

### 3.4 Sand temperature in beach, deforested, and hatchery environments

Sand temperatures at all logger locations exhibited temporal and spatial variation at Bahía de Jiquilisco (2012–2015) and Estero Padre Ramos (2015). At Bahía de Jiquilisco, temperatures steadily decreased by 1–2°C over the hawksbill nesting season at 30‐ and 60‐cm sand depths from April through October in beach zones and deforested areas, and from May through October in hatcheries (Figure 4a,c). Beach and hatchery sand temperatures at Estero Padre Ramos decreased from 33°C to 29°C in June, rose to near‐initial levels during July through August, and then decreased by 1–2°C from September through October (Figure 4b,d).

Mean sand temperatures during the nesting season at Bahía de Jiquilisco were greater than the most conservative male‐producing pivotal temperature estimate for hawksbills among studied hawksbill populations (29.7°C; Godfrey et al., 1999) in all nest environments at both sites, except woody vegetation and hatcheries (Figure 4e). Woody vegetation was >3°C cooler than open sand at Bahía de Jiquilisco and nonwoody vegetation at Estero Padre Ramos (Figure 4e,f). Deforested areas and woody vegetation at Bahía de Jiquilisco logged the highest (31.9 ± 1.7°C, *n* = 2,558 days) and lowest (28.5 ± 0.8°C, *n* = 853) mean seasonal temperatures, respectively, with hatchery temperatures falling between these values (29.6 ± 1.0°C, *n* = 1514; Figure 4e). Nonwoody vegetation and woody vegetation at Estero Padre Ramos had the highest (33.4 ± 1.1°C, *n* = 170) and lowest (30.2 ± 1.0°C, *n* = 170) seasonal mean temperatures, respectively, with intermediate hatchery temperatures (31.1 ± 1.0°C, *n* = 170; Figure 4f).

There were significant differences in temperature between sand depths in woody vegetation, deforested areas, and hatcheries and among years at Bahía de Jiquilisco (Table 5), with the 60‐cm depth warmer than the 30‐cm depth in woody vegetation (30 cm, 28.5 ± 0.9°C, *n* = 851 days; 60 cm, 28.6 ± 0.7°C, *n* = 768) and hatcheries (30 cm, 29.3 ± 0.9°C, *n* = 1,319; 60 cm, 29.8 ± 1.0°C, *n* = 1514; Figure 4e). Similarly, at Estero Padre Ramos, we detected significant differences in temperature between sand depths in nonwoody vegetation, woody vegetation, and the hatchery (Table 5), with the 60‐cm depth warmer than the 30‐cm depth in woody vegetation (30 cm, 30.0 ± 1.1°C, *n* = 170; 60 cm, 30.4 ± 0.9°C, *n* = 170) and the hatchery (30 cm, 30.7 ± 1.0°C, *n* = 170; 60 cm, 31.5 ± 0.7°C, *n* = 170; Figure 4f). In all nest environments, fluctuations in daily temperature were greater at the 30‐cm than at the 60‐cm depth, regardless of mean daily temperature (Figure 4a–d).

**Table 5:** Two‐way ANOVA results for differences in sand temperature between logger depths (30 and 60 cm), among years, and with interactions for each of six nest environments at Bahía de Jiquilisco, El Salvador, 1 April to 31 May 2012–2015, and between logger depths for each of four nest environments at Estero Padre Ramos, Nicaragua, 15 May to 31 October 2015

Nest environment source	Bahía de Jiquilisco					Estero Padre Ramos				
	*df*	SS	MS	*F*	*p*	*df*	SS	MS	*F*	*p*
Open sand										
Depth	1	16.3197	16.3197	2.3398	0.1262	–	–	–	–	–
Year	3	26.4532	8.8177	1.2642	0.2849	–	–	–	–	–
Depth × Year	3	28.2006	9.4002	1.3477	0.2571	–	–	–	–	–
Error	2,946	20,547.6360	6.9748			–	–	–	–	–
Total	2,953	20,694.7090	7.0080			–	–	–	–	–
Nonwoody										
Depth	1	0.1221	0.1221	0.1097	0.7405	1	5.1171	5.1171	4.3643	0.0374
Year	3	320.5849	106.8616	96.0888	<0.0001	–	–	–	–	–
Depth × Year	3	3.7951	1.2650	1.1375	0.3326	–	–	–	–	–
Error	1655	1840.5473	1.1121			338	396.3100	1.1725		
Total	1662	2,424.9954	1.4591			339	401.4272	1.1842		
Woody border										
Depth	1	1.9467	1.9467	0.7614	0.3829	1	0.8875	0.8775	0.4537	0.5010
Year	3	1598.5779	532.8593	208.4220	<0.0001	–	–	–	–	–
Depth × Year	3	68.2353	22.7451	8.8965	<0.0001	–	–	–	–	–
Error	3,188	8,150.5590	2.5566			338	653.6386	1.9338		
Total	3,195	11,174.2280	3.4974			339	654.5161	1.9307		
Woody										
Depth	1	4.9666	4.9666	10.1207	0.0015	1	14.1739	14.1739	14.8321	0.0001
Year	3	179.1777	59.7259	121.7070	<0.0001	–	–	–	–	–
Depth × Year	3	2.6335	0.8778	1.7888	0.0679	–	–	–	–	–
Error	1612	791.0646	0.4907			338	323.0011	0.9556		
Total	1619	1,107.1076	0.6838			339	337.1750	0.9946		
Deforested										
Depth	1	152.6609	152.6609	35.9149	<0.0001	–	–	–	–	–
Year	3	1,328.3203	442.7734	104.1666	<0.0001	–	–	–	–	–
Depth × Year	3	29.6735	9.8912	2.3270	0.0726	–	–	–	–	–
Error	4,684	199,909.9400	42.6793			–	–	–	–	–
Total	4,691	22,312.8140	4.7565			–	–	–	–	–
Hatchery										
Depth	1	115.7045	115.7045	159.2262	<0.0001	1	63.1152	63.1152	85.7970	<0.0001
Year	3	195.0369	65.0123	89.4663	<0.0001	–	–	–	–	–
Depth × Year	3	16.2924	5.4308	7.4736	<0.0001	–	–	–	–	–
Error	2,825	2,052.8360	0.7292			338	248.6444	0.7356		
Total	2,832	2,808.2782	0.9916			339	311.7596	0.9196		

## 4 DISCUSSION

Uncertainty exists regarding the ability of long‐lived thermally sensitive reptiles, such as sea turtles, to exhibit compensatory responses to accelerated climate‐driven environmental changes capable of offsetting negative consequences to population demographics (Hays, Mazaris, Schofield, & Laloë, 2017; Laloë, Cozens, Renom, Taxonera, & Hays, 2017). For eastern Pacific hawksbills nesting in mangrove estuaries at Bahía de Jiquilisco, El Salvador, and Estero Padre Ramos, Nicaragua, our results demonstrate that clutches protected in situ incubated at higher temperatures, yielded lower hatching success, produced a higher percentage of female hatchlings, and produced less fit offspring than clutches relocated to hatcheries. Additionally, sand temperature data of nesting beaches indicate that most nest environments already surpass the pivotal temperature for hawksbills, with higher temperatures at the deeper depth in the coolest nest environments (i.e., woody vegetation and hatchery).

### 4.1 Natural nests produce fewer males and less fit hatchlings

Hawksbill clutches incubated in beaches within mangrove estuaries at Bahía de Jiquilisco and Estero Padre Ramos had relatively low hatching success (56.8%) across all protection strategies compared to hawksbill nesting on open‐coast beaches in the eastern Pacific (e.g., 64.5%, Gaos et al., 2017), Caribbean (e.g., 91.6%, Bjorndal, Carr, Meylan, & Mortimer, 1985; 84.5%, Horrocks & Scott, 1991; 78.6%, Ditmer & Stapleton, 2012), and Indo‐Pacific (90.1% [emergence success], Limpus, 1980; 79.9% [emergence success], Loop et al., 1995; 82.4%, Dobbs, Miller, Limpus, & Landry, 1999; 85.2%, Hoenner et al., 2016). We suspect differences in overall hatching success reflect distinct biophysical conditions of beaches in mangrove estuaries, such as presence of extremely fine‐grained sand. Because sand grain size affects water and gas flux (Ackerman, 1980), sand consisting of small particle sizes could have interstitial spacing and high water content that inhibits respiratory gas exchange of developing embryos (Ackerman, 1997), which could lower hatching success. For example, nesting beaches at Bahía de Jiquilisco consist of a high proportion (90.1%) of sand particle sizes measuring ≤0.125 mm (Y. Flores, unpublished data), which is substantially smaller than sand grain sizes reported for hawksbill nesting beaches in other geographic regions (Ditmer & Stapleton, 2012; Dobbs et al., 1999; Zare, Vaghefi, & Kamel, 2012).

We found significantly lower hatching success in clutches protected in situ (43.6%) than clutches relocated on the beach (46.2%) or in hatcheries (58.6%) at both sites (Table 4). This difference probably arises primarily from differences in microenvironmental conditions during incubation (Eckert & Eckert, 1990; Kornaraki et al., 2006; Revuelta et al., 2015), such as the amount of organic content (e.g., roots and leaves) in the sand, which is likely lower in hatcheries due to removal of organic material during hatchery preparation. This is consistent with hawksbill clutches in Antigua (Caribbean), where hatching success increased as a function of decreasing organic content in the sand (Ditmer & Stapleton, 2012). Hatchery preparation processes could further favorably alter conditions of nest environments by lowering sand compaction within the hatchery enclosure, which could facilitate respiratory gas exchange of developing embryos (Garrett, Wallace, Garner, & Paladino, 2010).

We estimate that hawksbill nesting beaches produced 66.0%–81.0% female hatchlings across nest protection strategies at our sites, with a slightly higher percentage of females produced at Bahía de Jiquilisco than Estero Padre Ramos (Table 2). Our results represent lower female‐biased sex ratios than reported at many sea turtle nesting beaches in other ocean basins (Hawkes et al., 2009; Poloczanska, Limpus, & Hays, 2009; Wibbels, 2003), but female production was more pronounced in clutches protected in situ, with 88.9%–96.2% and 68.1%–88.3% females at Bahía de Jiquilisco and Estero Padre Ramos, respectively. Clutches relocated to hatcheries at Estero Padre Ramos experienced a significant shift in sex ratios from highly male‐biased in 2010–2011 to highly female‐biased in 2012–2015 (Figure 3d). This shift is likely due to a change in hatchery location from a site with 100% overstory vegetation cover to an area with less cover (77.7%; Table 1), combined with climatic factors—such as cooler ambient temperature and increased precipitation associated with La Niña—reflected by longer incubation durations across protection strategies at Estero Padre Ramos. We attribute the higher percentage of female hatchlings produced at Bahía de Jiquilisco primarily to the degraded condition of coastal forest at many beaches relative to the higher‐quality habitat that is available to nesting turtles at Estero Padre Ramos (Liles, Peterson, Seminoff, et al., 2015b), including areas where clutches are protected in situ. Indeed, vegetation cover can predict nest temperatures (Kamel, 2013) and hatchling sex (Janzen, 1994), which highlights the importance of preserving and restoring natural vegetation cover at hawksbill nesting beaches.

Hatchling length and mass differed among nest protection strategies and among years (Table 4), with hatchlings that were smaller and weighed less from clutches protected in situ than clutches relocated on the beach or in hatcheries (Table 2). Previous studies indicate that nest temperature is inversely correlated with hatchling body size, where warmer nests produce hatchlings with smaller carapaces and flippers, but that nest temperature did not influence hatchling mass (Booth, Feeney, & Shibata, 2013; Maulany, Booth, & Baxter, 2012; Wood, Booth, & Limpus, 2014). Hatchlings with larger carapaces and flippers are likely to crawl faster and employ more thrust while swimming than smaller hatchlings (Ischer, Ireland, & Booth, 2009; Janzen, Tucker, & Paukstis, 2000), which may allow them to more quickly navigate away from near‐shore predators to offshore waters and thus increase their chance of survival (Booth, 2017; Wood et al., 2014).

It is unclear, however, whether increased carapace size and locomotor performance in hatchlings at open‐coast beaches confer similar advantages to hatchlings at inshore beaches in mangrove estuaries. Ongoing research into dispersal patterns of hawksbill hatchlings in Bahía de Jiquilisco suggests that hatchling movements are regulated by tidal currents in the estuary, where turtles tend to passively drift camouflaged among floating debris (e.g., mangrove shoots and leaves) while transported by tidal currents (M. Liles, unpublished data). This behavior suggests that smaller hatchlings from warmer in situ nests may not necessarily be at a comparative disadvantage to larger hatchlings from clutches relocated on the beach and in hatcheries while inside mangrove estuaries, but could be at a disadvantage if transported outside the estuary and thence required to actively swim to encounter ocean currents.

### 4.2 Warmer sand temperatures at the deeper depth

Our data on seasonal sand temperature in nest environments delineate temporal and spatial differences in hawksbill nesting environments at Bahía de Jiquilisco and Estero Padre Ramos. We found sand temperatures generally decreased from ocean to forest, with woody vegetation and hatcheries cooler than other nest environments (Figure 4e,f), which is consistent with thermal patterns reported for some hawksbill nesting beaches (Kamel, 2013; Kamel & Mrosovsky, 2006a), but contrasts with studies at other hawksbill nesting beaches that detected no difference between unshaded and shaded areas (Glen & Mrosovsky, 2004; Mrosovsky, Bass, Corliss, Richardson, & Richardson, 1992).

For most beach and hatchery environments at Bahía de Jiquilisco and Estero Padre Ramos, mean sand temperature was higher at the deeper depth (Figure 4e,f), which contrasts with the prevailing paradigm that temperatures are lower at deeper depths (Glen & Mrosovsky, 2004; Hill, Paladino, Spotila, & Santidrián Tomillo, 2015; Naro‐Maciel et al., 1999). For example, Laloë, Esteban, Berkel, and Hays (2016) found consistently cooler sand temperature at deeper depths along a hawksbill nesting beach on St. Eustatius Island (Caribbean), where temperature at 100 cm was 1°C cooler than at 40–60 cm. One potential explanation for warmer temperatures at the deeper depth at our sites is the presence of groundwater at a depth of <1 m during the nesting season. Because groundwater absorbs and redistributes geothermal heat as it flows horizontally (Cartwright, 1974), the temperature of shallow groundwater (<10 m) can be 1–2°C greater than the mean annual surface temperature (Anderson, 2005) which can be further amplified in heavily shaded areas (Lewis & Wang, 1998), such as in woody vegetation and hatchery environments at our sites (Figure 4e,f).

### 4.3 Potential limitations of behavioral plasticity in climate change adaptation

Previous studies argue that sea turtles may adapt to climate change through nesting behavioral plasticity, including redistribution of nesting ranges (Limpus, 2006; Pike, 2013b; Schofield et al., 2010), shifts in nesting phenology toward cooler months (Patel et al., 2016; Saba, Stock, Spotila, Paladino, & Santidrián Tomillo, 2012; Weishampel, Bagley, & Ehrhart, 2004), changes in nest‐site selection (Hawkes et al., 2007; Hays et al., 2001), and alteration of nest depth (Hays et al., 2001; Laloë et al., 2016; Pike, 2013a). However, our findings indicate that mangrove ecosystems of Bahía de Jiquilisco and Estero Padre Ramos present a number of biophysical and human‐induced constraints that, when coupled with unique life‐history characteristics of eastern Pacific hawksbills, may limit behavioral compensatory responses by the species to projected temperature increases at nesting beaches.

Because >80% of female hawksbills in the eastern Pacific nest along low‐relief beaches on islands and peninsulas within mangrove estuaries (Gaos et al., 2017; Liles, Peterson, Seminoff, et al., 2015b), climate‐driven sea‐level rise threatens viability of current nesting beaches. Global mean sea level is projected to rise between 0.26 and 0.98 m (Church, Clark, & Cazenave, 2013), but to as high as 1.14 m when accounting for Greenland and Antarctica ice loss (DeConto & Pollard, 2016), by 2100. Under sea‐level rise scenarios of 0.1, 0.5, and 0.9 m, Fish et al. (2008) estimated that 4%, 26%, and 51% of total beach area, respectively, would be submerged from 11 low‐elevation beaches (1.25–3.09 m) with gentle slope (1.8–5.8°) used by nesting hawksbills on Barbados (Caribbean), with similar estimates (i.e., 14%, 31%, and 51%, respectively) for 13 low‐relief hawksbill nesting beaches on Bonaire (Caribbean; Fish et al., 2005). Given that most hawksbill nesting beaches at Bahía de Jiquilisco and Estero Padre Ramos have an elevation of ≤1 m above mean sea level with marginal slope (<2°), beach loss of 4%–51% likely represents a conservative estimate for our sites under sea‐level rise scenarios of 0.1–0.9 m by 2100. Indeed, vulnerability of nesting beaches to sea‐level rise was exemplified by a flooding event that occurred at Bahía de Jiquilisco in 2015, where all eight nesting beaches were temporarily inundated from extraordinarily high tides and precipitation, resulting in total mortality of 30 hawksbill clutches.

Strategies to mitigate beach loss from climate change include enforcement of existing construction setback regulations and prevention of coastal infrastructure that alter nesting areas (Fuentes, Fish, & Maynard, 2012). Although conservation setbacks can be an important tool for maintaining nesting beach integrity (e.g., Fish et al., 2008), and despite nominal protective measures that prohibit human use of beaches 100 m landward from the high tide line in Nicaragua (República de Nicaragua, 2009), most beaches at our sites are backed by human settlements, small‐scale agriculture, or mangrove forests in the intertidal zone, which can restrict inland retreat of beaches. While the paleoenvironmental record indicates that mangroves have adjusted to sea‐level changes over millennia through vertical sediment accretion and subsurface root accumulation (Ellison, 2008; Woodroffe et al., 2016), the current rate of sea‐level rise likely will outpace gain in soil surface elevation, and in areas where physical barriers (e.g., aquaculture ponds, coastal infrastructure, and agricultural fields) prevent landward migration, such as at our sites, mangroves may submerge (Lovelock et al., 2015). Given that 90% (*n* = 564 clutches annually) of hawksbill reproductive output in the eastern Pacific is concentrated at five nesting sites within only one degree latitude (12°35′–13°35′N; Gaos et al., 2017), highly specific biophysical (e.g., sand morphology and ocean currents) and human‐induced (e.g., depredation and beach development) conditions govern viability of these areas as suitable nesting habitat, suggesting that latitudinal redistribution to exploit other Central American beaches where similar climatic patterns are projected to occur seems unlikely (Saba et al., 2012; Santidrián Tomillo et al., 2012).

Shifts in nesting phenology have been observed for some sea turtle populations (Azanza‐Ricardo et al., 2017; Patel et al., 2016; Weishampel et al., 2004). Because sand temperatures at Bahía de Jiquilisco and Estero Padre Ramos generally decreased over the nesting season in all nest environments at both depths (Figure 4a–d), the decrease in temperature between the beginning (April–May) and end (September–October) of the nesting season—which is reflected in shorter incubation durations and higher percentage of female hatchlings produced during the first half than the second half of the nesting season (Figure 3a,b)—suggests that hawksbills could respond to projected temperature increases by nesting later in the season to exploit cooler temperatures. Additionally, turtles that currently nest in September–October at both sites may have an adaptive advantage (Valladares et al., 2014), highlighting the importance of protecting the nests of these individuals, even if their numbers are relatively fewer during those later months.

Some turtle populations appear to be capable of spatially adapting nest placement to align the current thermal niche of the nest environment with changing climatic conditions, whereas others seem relatively inflexible. For example, female painted turtles from five distinct populations across their geographic range that were translocated to a common environment differed in choice of nesting date and nest depth, but did not differ in shade cover, resulting in similar incubation regimes across populations despite differences in local climate at their locations of origin (Refsnider & Janzen, 2012). In contrast, individual hawksbills in the Caribbean are highly consistent in their nest microhabitat preferences, including vegetative cover above nests within and between years (Kamel & Mrosovsky, 2005, 2006b), suggesting that female hawksbills are relatively constrained in their ability to alter nesting behavior (Kamel, 2013). Although hawksbills at Bahía de Jiquilisco and Estero Padre Ramos are highly consistent in their selection of vegetative cover, they exhibit locally specific adaptations shaped by microhabitat differences at each site (Liles, Peterson, Seminoff, et al., 2015b). For example, nest placement by hawksbills at Bahía de Jiquilisco is restricted to the narrow tract of secondary forest measuring 10–15 m wide adjacent to the high water line at most beaches, whereas nest placement at Estero Padre Ramos extends nearly twice the distance inland within intact second‐growth forest that is present >100 m landward from the high water line at most beaches (Liles, Peterson, Seminoff, et al., 2015b). Such adaptations may indicate the potential for development of compensatory responses to climate variability through nest‐site choice.

However, mangrove ecosystems are among the most threatened tropical environments in the world, with deforestation rates as high as 3.6% per year in the Americas (Valiela, Bowen, & York, 2001), suggesting that future degradation of forest habitat may impair its ability to buffer against increasing temperatures (Patrício et al., 2017). Coastal forests at our sites are confronted with the persistent threat of conversion by competing land uses, and forests along nesting beaches at Bahía de Jiquilisco have already experienced substantial alteration that restricts nest‐site selection by hawksbills (Liles, Peterson, Seminoff, et al., 2015b). Our findings suggest that inability to halt the continued fragmentation of intact woody vegetation will progressively replace cooler male hatchling producing refugia (28.5°C) for naturally incubating clutches with markedly warmer woody vegetation border (30.2°C) and deforested (31.9°C) areas, increasing the probability of highly female‐biased sex ratios (Poloczanska et al., 2009) and ultimately, climate‐driven egg and hatchling mortality (Santidrián Tomillo et al., 2012).

The ability of egg‐burying species to alter nest depth to compensate for increasing temperatures has been advanced as a possible adaptive strategy, presumably under the basic assumption that nest environments become cooler with increasing depth (Davenport, 1989; Fuentes & Porter, 2013). Our results, however, indicate that adjustment of nest depth by hawksbills is unlikely to compensate for climate change in mangrove estuaries. First, we detected higher temperatures at the deeper depth in most nest environments at both sites (Figure 4e,f). Second, the water table is at a depth of 50–85 cm during the nesting season at many beaches, which can be expected to become shallower as sea levels rise and further constrict suitable nest environments (Pike, 2014). This likely explains, at least in part, why hawksbills construct shallower nest cavities at Bahía de Jiquilisco (38.2 cm) and Estero Padre Ramos (40.5 cm) than at open‐coast nesting locations in the Caribbean (e.g., 47.0 cm, Kamel & Mrosovsky, 2006a), Indo‐Pacific (e.g., 45.3 cm, Loop et al., 1995), and Indian Ocean (e.g., 46.5 cm, Hitchins, Bourquin, Hitchins, & Piper, 2004). Finally, male hatchling production at Bahía de Jiquilisco and Estero Padre Ramos is not correlated with nest depth for clutches protected in situ, suggesting that shifts in nesting phenology and nest‐site choice may be more effective adaptive responses to a warming climate.

The accelerated rate at which climate change is projected to occur, together with other interacting anthropogenic threats, may outpace the biological capacity of sea turtles to adapt (Fuentes, Hamann, & Limpus, 2010). The inability of sea turtles to adaptively respond through behavioral or evolutionary mechanisms (e.g., adjust pivotal temperature; Davenport, 1989) would require that humans intervene to prevent local extinctions, such as watering, shading, and clutch relocation to modify sand temperatures and reduce egg and hatchling mortality (Hill et al., 2015; Jourdan & Fuentes, 2015; Wood et al., 2014). Indeed, we found that hawksbill clutches relocated on the beach and protected in shaded hatcheries had higher hatching success, produced higher proportions of male offspring, and produced fitter hatchlings than clutches protected in situ at Bahía de Jiquilisco and Estero Padre Ramos. However, we are not suggesting egg relocation as a panacea that should be employed without careful consideration of local conditions, species biology, and conservation objectives. Previous studies have highlighted negative consequences of hatcheries using poor management practices, such as low hatching success (Boulon, Dutton, & Mcdonald, 1996), biased sex ratios of hatchlings (Morreale, Ruiz, Spotila, & Standora, 1982), and increased hatchling mortality (Pilcher & Enderby, 2001). We contend, however, that egg relocation can contribute substantively to recovery efforts under appropriate circumstances. Our results underscore the importance of empirical assessments to evaluate potential mitigation strategies for severely depleted populations of highly endangered species that may be unable to respond sufficiently to climate change.

## CONFLICT OF INTEREST

None declared.

## AUTHOR CONTRIBUTIONS

MJL, TRP, JAS, ARG, BPW, and MJP conceived and designed the study. MJL, EA, AVH, VG, SC, and JU collected data. MJL and MJP carried out data analyses. MJL led writing of the manuscript with input and critical review from all authors.

## Data Availability

Hawksbill hatchling and temperature data can be accessed in the Dryad Digital Repository https://doi.org/10.5061/dryad.33rq371.
